# Genetic variability in the *PLIN4* gene: A new sequence duplication causing autophagic vacuolar myopathy

**DOI:** 10.1016/j.gendis.2025.101849

**Published:** 2025-09-10

**Authors:** Alessandra Carnazzi, Eliana Iannibelli, Sara Gibertini, Lucia Nicolini De Gaetano, Giorgia Riolo, Franco Salerno, Andrea Legati, Daniele Ghezzi, Lorenzo Maggi, Alessandra Ruggieri

**Affiliations:** a Neuroimmunology and Neuromuscular Disease Unit, Fondazione IRCCS Istituto Neurologico Carlo Besta, Milan 20133, Italy; b Department of Pharmacological and Biomolecular Sciences, University of Milan, Milan 20133, Italy; c Medical Genetics and Neurogenetics Unit, Fondazione IRCCS Istituto Neurologico Carlo Besta, Milan 20133, Italy; d Department of Pathophysiology and Transplantation, University of Milan, Milan 20122, Italy

*PLIN4*-related myopathy is a rare autosomal dominant disorder first described in 2020 in a large Italian family,^1^ presenting with weakness of lower distal limbs often combined with upper distal limbs, and with scapular and pelvic muscle as the predominant pattern of muscle involvement, without any relevant cardiac or respiratory implications.^2^ This myopathy was linked to a pathogenic expansion in the *PLIN4* gene,^1^ encoding for perilipin 4 protein, whose function in the muscle is still unknown. This possesses an amphipathic region composed of 31 highly similar but not identical repeats, each of them formed by 99 nucleotides, corresponding to 33 amino acids.^3^ The reported mutation affects the highly repetitive exon 5 of the gene, and is due to the expansion of a single repeat increasing the number of 33-amino acid repeats from 31 to 40 (9 extra repeats).^1^ This expansion results in the accumulation of perilipin 4 in the subsarcolemmal region of the myofibers and within the vacuoles, with activation of the specialized aggrephagy pathway for the degradation of protein aggregates through autophagy. Over time, the elimination of accumulated vacuoles likely becomes a challenge, as vacuoles continue to form and fuse with each other, compromising the spatial organization of the fibers. This prevents the fibers from contracting properly, thus leading to their degeneration.

This family represents the largest cohort to date,^2^ but three other unrelated families and one sporadic patient were recently reported in China.^4^^,^^5^ All these cases showed the expansion of the same 33-amino acid repeat as originally reported, with associated activation of the aggrephagy pathway. Interestingly, one of these families with a longer expansion of the same repeat (19 additional repeats, 50 repeats in total) was associated with a more severe phenotype and an earlier presentation.^5^

In this study, we report the case of a male Italian patient presenting with proximal lower limb muscle weakness associated with difficulty in climbing stairs at the age of 59 years. Family history was negative for neuromuscular disorders, although no information is available in the paternal ancestors. He has only one daughter, now 39 years old, reported as asymptomatic, but never clinically and genetically investigated. Notably, an episode of severe generalized weakness after intense exercise occurred at the age of 14 years, followed by spontaneous full recovery. Serum creatine kinase levels persistently increased (range 1000–2166 U/L) over time, values that were never reported before. Electromyography at the age of 60 years revealed mild diffuse myopathic changes and myotonic discharges at the trapezius and quadriceps muscles. The patient underwent a first muscle biopsy at the right quadriceps at the age of 60 years in another hospital, which revealed myopathic changes associated with rimmed vacuoles in 3–4 fibers. Muscle MRI at the age of 62 years showed complete adipose tissue replacement of the soleus, adductor magnus, and long head of biceps femoris and semimembranosus, with partial fatty substitution of semitendinosus, lumbar paravertebral, gluteus medius, and minimus muscles (Fig. 1A–C). Neurological examination at our institute at the age of 66 years showed waddling gait, Gowers' manoeuvre, moderate weakness of pelvic muscles, and mild weakness of long finger extensors and scapular muscles, with evidence of scapular winging (Fig. 1D). No cranial or axial muscle weakness was observed. A second muscle biopsy of the left quadriceps was performed at the age of 66 years, revealing frequent hypotrophic fibers, internalized nuclei, frequent lobulated fibers, marked type I fiber predominance, and rare rimmed vacuoles. The patient reported a slow progression of muscle weakness over the years. Neurological examination at the age of 69 years showed minimal worsening of pelvic muscle weakness. Inclusion body myositis-functional rating scale score was 35/40, hence quite preserved (normal value 40/40). Spontaneous or percussion myotonia was not detected. He never complained of bulbar symptoms. The patient also suffered from chronic obstructive pulmonary disease, needing nocturnal non-invasive ventilation since the age of 65 years, and limiting his walking ability to 20–30 m due to breathing difficulties.

**Figure 1:**
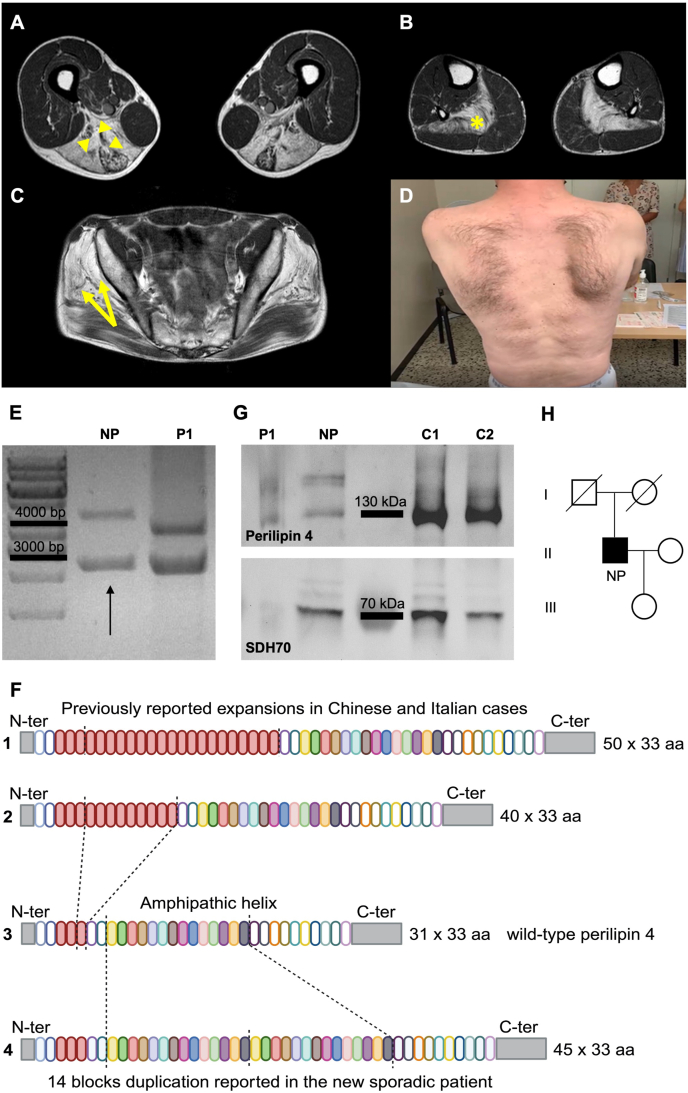
Clinical and molecular features of the novel pathogenetic *PLIN4* mutation. **(A**–**C)** Representative images of (A) thigh, (B) leg, and (C) pelvis sections on axial T1W images showing fatty substitution of gluteus medius and minimus muscles (arrows), adductor magnus, long head of biceps femoris and semimembranosus (arrowheads), and soleus (∗). **(D)** Winging of the right scapula during arm flexion. **(E)** PCR amplification of exon 5 showing a second higher band corresponding to the mutated allele in the new patient (NP), compared with a patient from the original family (P1). **(F)** Schematic representation of the pathogenic variants affecting the amphipathic region of perilipin 4: (1) and (2) showing the already reported expansions increasing the number of 33 amino acid repeats to 50 and 40, respectively; (3) wild-type perilipin 4 with 31 repeats of 33 amino acids; (4) the novel pathogenetic mutation characterized by a duplication of 14 repeats, producing a final protein with 45 repeats in the amphipatic region. **(G)** Western blots of biopsy-derived protein extracts confirming the presence of a larger protein in the new patient (NP) compared with patient P1 reported by Ruggieri et al in 2020 and the controls (C1 and C2). **(H)** Family pedigree.

Routine diagnostic analysis by targeted next-generation sequencing (Table S1), using the HaloPlex Target Enrichment System (Agilent Technologies) for Illumina MiSeq sequencing, did not reveal any pathogenic variants in genes commonly involved in myopathies. Based on the histopathology of the muscle biopsy, we investigated a possible involvement of *PLIN4*. We thus performed a PCR amplification of the patient's DNA with specific primers for exon 5^1^ followed by gel electrophoresis analysis. This showed the presence of a second band corresponding to the mutated allele, whose electrophoretic migration was revealed to be slower than the already described expanded allele, hinting at a larger rearrangement (Fig. 1E).

To characterize this rearrangement, we performed long-read sequencing analysis with Oxford Nanopore technology. PCR products were processed for library preparation with the Ligation Sequencing Kit (SQK-LSK109) protocol (Nanopore Community), adapter-ligated, and thus loaded into the MinION Flow Cell R9.4.1. The resulting raw reads were firstly directly aligned to the human reference genome (assembly GRCh37 (hg19)), and statistical analyses were performed, including the read length calculation of reads mapping on chromosome 19.

We were thus able to identify the most represented read population, performing an *in silico* gel to separate the wild-type (lower) band with 31 repeats from the mutant (higher) one carrying the repeat expansion. Given the unknown nucleotide composition of this expansion, the human reference genome was not suitable for further characterization of the repeat expansion. Accordingly, a *de novo* reference genome was generated, and raw reads belonging to each virtual band were re-aligned (see supplementary data for details).

In particular, long-read sequencing revealed a duplication of 1386 nt encoding for 462 aa (14 repeats), corresponding to aa 491 to aa 953 of the patient's wild-type allele (Fig. S1). This is a different rearrangement compared with the already reported mutations, which involve a different repeat (aa 326 to aa 359) (Fig. 1F). The presence of a bigger protein encoded by this duplication was confirmed by western blotting analysis of biopsy-derived protein extracts using perilipin 4 antibody (Fig. 1G).

Furthermore, in line with the observations in the previous cases, histological evaluation of the patient's muscle biopsy showed staining positive of autophagic vacuoles and subsarcolemmal regions for p62/SQSTM1 and NBR1, and the ubiquitinated protein marker FK2. All these proteins co-localized with perilipin 4, supporting our hypothesis of aggrephagy activation as an attempt to remove the protein aggregates. Moreover, when staining for two other aggrephagy-related markers, Toll-like interacting protein (TOLLIP) and optineurin, we detected positivity within the vacuoles for the first one and in the subsarcolemmal region of some fibers as well as in the vacuoles for the latter (Fig. S2).

Strikingly, our case shows a unique correlation between genetic rearrangements in the *PLIN4* gene and the severity of the phenotype. In fact, one family including two patients reported by Yang et al carried a longer genetic expansion and displayed higher disease severity and earlier onset compared with the other reported cases (19 *vs*. 9 extra repeats), suggesting a genotype–phenotype correlation with the size of the rearrangement.^2^^,^^4^^,^^5^ However, our patient, despite having a larger mutant protein than the cases carrying 9 extra repeats, shows a relatively mild phenotype, particularly concerning the disease duration and age at onset. Of note, the duplication found in our new patient involves a completely different part of exon 5, encoding for distinct amino acids. This indicates that the severity of the resulting disease could be influenced not only by the number of additional repeats and the total size of the resulting protein, but also by the amino acid composition of the involved repeats.

In conclusion, our report shows that the highly repetitive *PLIN4* gene can undergo different pathogenic rearrangements, particularly in exon 5, activating the same aggrephagy pathway, yet giving rise to different phenotypic manifestations. This characteristic makes *PLIN4*-related myopathies challenging to diagnose and suggests that more patients with *PLIN4* pathogenic variants are likely still to be discovered.

## CRediT authorship contribution statement

**Alessandra Carnazzi:** Writing – original draft, Methodology, Validation, Formal analysis, Conceptualization. **Eliana Iannibelli:** Methodology, Validation, Writing – review & editing. **Sara Gibertini:** Writing – review & editing, Methodology. **Lucia Nicolini De Gaetano:** Writing – review & editing, Methodology. **Giorgia Riolo:** Writing – review & editing, Methodology. **Franco Salerno:** Methodology, Writing – review & editing. **Andrea Legati:** Writing – review & editing, Methodology, Software. **Daniele Ghezzi:** Methodology, Software, Writing – review & editing. **Lorenzo Maggi:** Validation, Supervision, Writing – original draft, Conceptualization. **Alessandra Ruggieri:** Supervision, Writing – original draft, Conceptualization, Methodology, Validation.

## Ethics declaration

This study was approved by the Institutional Review Board at the Fondazione IRCCS Istituto Neurologico C. Besta (Project identification code 83/2022) in compliance with the current version of the Declaration of Helsinki as well as all national legal and regulatory requirements. Patient provided informed written consent.

## Funding

This work was partially supported by the 10.13039/501100003196Italian Ministry of Health (RRC), and was supported by AFM Téléthon (SR 2024 #289888).

## Conflict of interests

All authors have no relevant financial or non-financial interests to disclose related to this work. However, L.M. received funding for travel, meeting attendance, and advisory board participation from Sanofi Genzyme, Roche, Biogen, Amicus Therapeutics, Alexion Pharmaceuticals, Janssen, UCB, Lupin, and Argenx. He also received funding for a fellowship from Biogen and Alexion Pharmaceuticals.
