# Supplementary material for: Genetic variability in the PLIN4 gene: A new sequence duplication causing autophagic vacuolar myopathy
Source: Genes Dis. 2025 Sep 10;13(3):101849. doi: 10.1016/j.gendis.2025.101849 (PMC12854863; doi:10.1016/j.gendis.2025.101849)
Supplement: Multimedia component 1 [file mmc1.docx]

**SUPPLEMENTAL MATERIAL**

**Methods**

**Genetic analysis**

***ONT (Oxford Nanopore Technologies) Library Preparation and Sequencing***

Genomic DNA was amplified using a Phire Hot Start II DNA Polymerase (Thermo Fisher) optimized for long DNA fragments and specific primers for exon 5 (Fw-ACCACTCGGTCTGCACTTA and Rw-CCTCCAACTCATTCTGCAGC) according to the protocol previously described [1]. The PCR products were processed by the Ligation Sequencing Kit (SQK-LSK109) protocol (Nanopore Community [https://nanoporetech.com]). In detail, the library preparation started with the DNA ends repairing for the further adapter attachment step. After purifying the reaction mix with AMPure XP beads, the repaired and end-prepped DNA was ready for the sequencing adapters to be attached to DNA ends. The clean-up step after adapter ligation was performed using the wash buffer LFB (Long Fragment Buffer). Once the DNA library was adapter-ligated, it was loaded into the MinION Flow Cell R9.4.1.

***Long-reads Downstream Bioinformatic Analysis***

Raw reads, resulting from the real time MinKNOW software data acquisition, were directly aligned to the reference genome (Homo sapiens (human) genome assembly GRCh37 (hg19)) using Minimap2 [2]. We performed minimap2 alignment taking into account spliced long-reads (unknown strand) and aligning them to the reference genome file. In order to visualize the alignment, we exploited the Integrative Genomics Viewer (IGV), to appreciate coverage depth and potential genome structural alterations [3].

***In silico gel and De Novo reference generation***

Statistical analyses were performed, including the read length calculation of reads mapping on chromosome 19.

In this way, we were able to identify the most represented read population, performing an “in silico” gel to separate the wild type (lower) and the band carrying the expansions (higher). Since the human reference genome was not suitable for the investigation of this complicated region, we proceeded generating a De Novo reference genome with Canu, a tool to assemble long reads from either PacBio or ONT [4].

***Second Alignment using De Novo reference sequence and coverage visualization.***

Raw reads belonging to each virtual band were re-aligned to the De Novo reference fasta sequence of the highest band obtained by Canu, using Minimap2. The produced alignments were visualized by IGV software.

**Western blot analysis**

Muscle tissue fragments were processed as previously reported [1] and run on a 7% acrylamide gel to visualize the presence of a higher second band encoded by the mutated allele. Primary antibodies used were perilipin 4 (guinea pig polyclonal AP33136SU-N, OriGene, Rockville, USA) and SDH70 (anti-complex II 70 kDa Fp subunit monoclonal antibody, cod 459200, Invitrogen, Thermo Fisher Scientific, Carlsbad, California, USA) as loading control.

**Immunofluorescence analysis**

Histological evaluation of patient’s muscle biopsy by immunostaining was performed as previously described [1] to show the activation of the aggrephagy pathway for the degradation of protein aggregates through autophagy [5; 6]. Specifically, transverse cryostat sections were stained for perilipin 4 (guinea pig polyclonal GP34, Progene Biotechnik, Heidelberg, Germany; 1:100), poly-ubiquitinylated proteins (mouse monoclonal PW8810, clone FK2 from Biomol, Enzo Life Sciences, Inc. Farmingdale, USA; 1:100), p62 (guinea pig polyclonal GP62-c, Progene Biotechnik, Heidelberg, Germany; 1:100), NBR1 (mouse monoclonal 4BR, Santa Cruz Biotechnology Inc, Santa Cruz CA, USA; 1:100), TOLLIP (rabbit polyclonal, PA5-83431, Invitrogen, Thermo Fisher Scientific, Carlsbad, California, USA; 1:50) and optineurin (mouse monoclonal, sc-166576, Santa Cruz Biotechnology Inc, Santa Cruz CA, USA; 1:50). Images were taken with a Nikon Eclipse Ti2 inverted microscope (Plan Apo 40x/1.0 Oil, Nikon Instruments Inc).

**Supplemental figure**


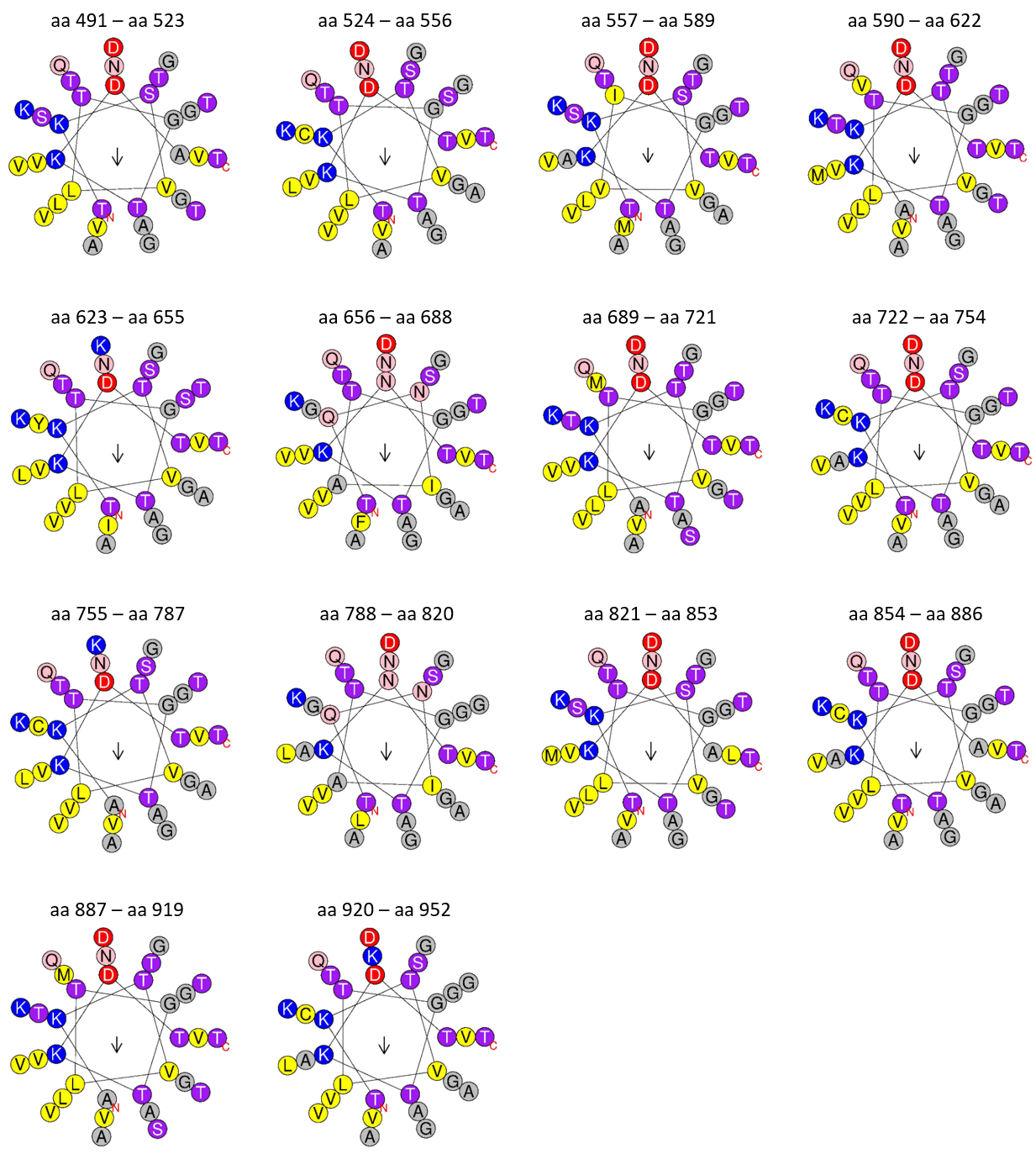


**Figure S1.** Schematic representation modeled with the HeliQuest web server [7] showing the duplicated amino acids organized in 3-11 alpha helix repeats.


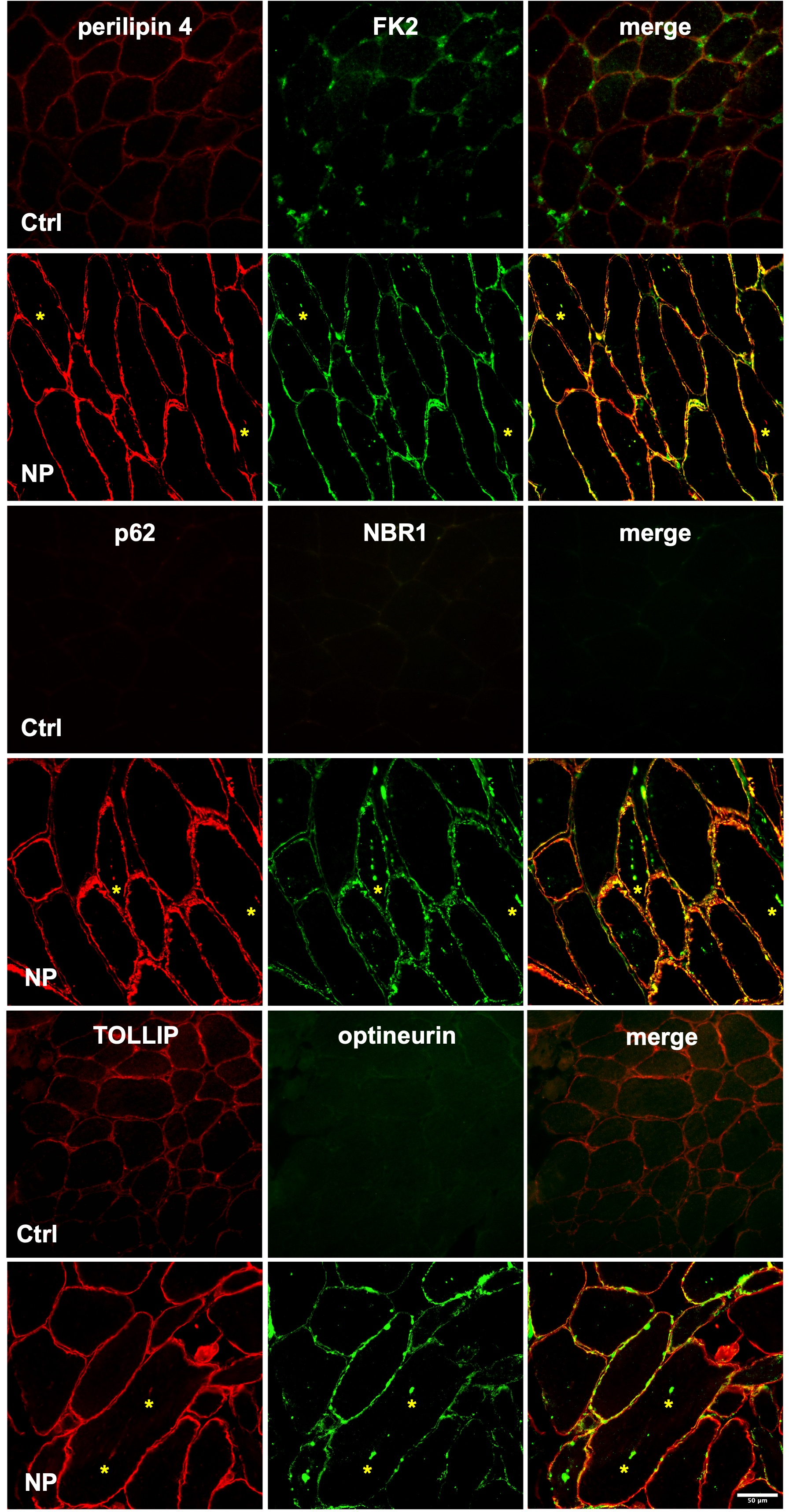


**Figure S2.** Immunofluorescence staining of control and patients’ muscle biopsies showing autophagic vacuoles (*) and subsarcolemmal regions stained positive for perilipin 4 and the autophagy markers p62/SQSTM1 and NBR1, and the ubiquitinated protein marker FK2 in the patient and not in the control. Increased positivity within the vacuoles was also observed for TOLLIP and positivity for optineurin was detected in the subsarcolemmal region of some fibers and within the vacuoles, unlike in the control (40x).

| **Table S1. List of genes included in the NGS myopathy gene panel** | | |
| --- | --- | --- |
| **Gene name** | **Chr location** | **Phenotype** |
| ACTA1 | 1q42.13 | Myopathy, scapulohumeroperoneal |
| ADSSL1 | [14q32.33](https://omim.org/geneMap/14/591?start=-3&limit=10&highlight=591) | Myopathy, distal, 5 |
| ANO5 | [11p14.3](https://omim.org/geneMap/11/243?start=-3&limit=10&highlight=243) | Miyoshi myopathy type 3 |
| BAG3 | [10q26.11](https://omim.org/geneMap/10/614?start=-3&limit=10&highlight=614) | Myopathy, myofibrillar, 6 |
| BICD2 | [9q22.31](https://omim.org/geneMap/9/310?start=-3&limit=10&highlight=310) | Distal myopathy |
| CASQ1 | [1q23.2](https://omim.org/geneMap/1/1315?start=-3&limit=10&highlight=1315) | Myopathy, vacuolar, with CASQ1 aggregates |
| CAV3 | [3p25.3](https://omim.org/geneMap/3/27?start=-3&limit=10&highlight=27) | Myopathy, distal, Tateyama type |
| CRYAB | [11q23.1](https://omim.org/geneMap/11/925?start=-3&limit=10&highlight=925) | Myopathy, myofibrillar, 2 |
| DES | [2q35](https://omim.org/geneMap/2/1040?start=-3&limit=10&highlight=1040) | Myopathy, myofibrillar, 1 |
| DNAJB6 | [7q36.3](https://omim.org/geneMap/7/862?start=-3&limit=10&highlight=862) | Distal myopathy |
| DYSF | [2p13.2](https://omim.org/geneMap/2/351?start=-3&limit=10&highlight=351) | Myopathy, distal, with anterior tibial onset |
| FHL1 | [Xq26.3](https://omim.org/geneMap/X/719?start=-3&limit=10&highlight=719) | Myopathy, X-linked, with postural muscle atrophy/ Scapuloperoneal myopathy, X-linked dominant |
| FLNC | [7q32.1](https://omim.org/geneMap/7/659?start=-3&limit=10&highlight=659) | Myopathy, distal, 4/ Myopathy, myofibrillar, 5 |
| GNE | [9p13.3](https://omim.org/geneMap/9/187?start=-3&limit=10&highlight=187) | Nonaka myopathy |
| HSPB8 | [12q24.23](https://omim.org/geneMap/12/858?start=-3&limit=10&highlight=858) | Myopathy, myofibrillar, 13, with rimmed vacuoles |
| KLHL9 | [9p21.3](https://omim.org/geneMap/9/92?start=-3&limit=10&highlight=92) | Early-onset distal myopathy |
| KY | [3q22.2](https://omim.org/geneMap/3/704?start=-3&limit=10&highlight=704) | Myopathy, myofibrillar, 7 |
| LDB3 | [10q23.2](https://omim.org/geneMap/10/356?start=-3&limit=10&highlight=356) | Myopathy, myofibrillar, 4 |
| LMNA | [1q22](https://omim.org/geneMap/1/1243?start=-3&limit=10&highlight=1243) | Cardiomyopathy, dilated, 1A |
| MATR3 | [5q31.2](https://omim.org/geneMap/5/516?start=-3&limit=10&highlight=516) | Distal myopathy with VCPDM |
| MEGF10 | [5q23.2](https://omim.org/geneMap/5/410?start=-3&limit=10&highlight=410) | Congenital myopathy 10A, severe variant/ Congenital myopathy 10B, mild variant |
| MYH2 | [17p13.1](https://omim.org/geneMap/17/226?start=-3&limit=10&highlight=226) | Congenital myopathy 6 with ophthalmoplegia |
| MYH7 | [14q11.2](https://omim.org/geneMap/14/92?start=-3&limit=10&highlight=92) | Laing distal myopathy/Cardiomyopathy, dilated, 1S/ Cardiomyopathy, hypertrophic, 1 |
| MYOT | [5q31.2](https://omim.org/geneMap/5/497?start=-3&limit=10&highlight=497) | Myopathy, myofibrillar, 3 |
| ORAI1 | [12q24.31](https://omim.org/geneMap/12/895?start=-3&limit=10&highlight=895) | Myopathy, tubular aggregate, 2 |
| PLEC | [8q24.3](https://omim.org/geneMap/8/621?start=-3&limit=10&highlight=621) | Congenital myopathy |
| PYROXD1 | [12p12.1](https://omim.org/geneMap/12/223?start=-3&limit=10&highlight=223) | Myopathy, myofibrillar, 8 |
| SEPN1 | [1p36.11](https://omim.org/geneMap/1/304?start=-3&limit=10&highlight=304) | Congenital myopathy 3 with rigid spine |
| SMCHD1 | [18p11.32](https://omim.org/geneMap/18/24?start=-3&limit=10&highlight=24) | Facioscapulohumeral muscular dystrophy 2, digenic |
| SQSTM1 | [5q35.3](https://omim.org/geneMap/5/837?start=-3&limit=10&highlight=837) | Myopathy, distal, with rimmed vacuoles |
| STIM1 | [11p15.4](https://omim.org/geneMap/11/103?start=-3&limit=10&highlight=103) | Myopathy, tubular aggregate, 1 |
| TCAP | [17q12](https://omim.org/geneMap/17/488?start=-3&limit=10&highlight=488) | Cardiomyopathy, hypertrophic, 25 |
| TIA1 | [2p13.3](https://omim.org/geneMap/2/333?start=-3&limit=10&highlight=333) | Welander distal myopathy |
| TRIM54 | [2p23.3](https://omim.org/geneMap/2/129?start=-3&limit=10&highlight=129) | Cardiac myopathy |
| TRIM63 | [1p36.11](https://omim.org/geneMap/1/312?start=-3&limit=10&highlight=312) | Cardiac myopathy |
| TTN | [2q31.2](https://omim.org/geneMap/2/823?start=-3&limit=10&highlight=823) | Myopathy, myofibrillar, 9, with early respiratory failure/Cardiomyopathy, dilated, 1G/Congenital myopathy 5 with cardiomyopathy |
| VCP | [9p13.3](https://omim.org/geneMap/9/160?start=-3&limit=10&highlight=160) | Inclusion body myopathy with early-onset Paget disease and frontotemporal dementia 1 |
| VMA21 | [Xq28](https://omim.org/geneMap/X/799?start=-3&limit=10&highlight=799) | Myopathy, X-linked, with excessive autophagy |

**References**

1. Ruggieri A, Naumenko S, Smith MA, et al. Multiomic elucidation of a coding 99-mer repeat-expansion skeletal muscle disease. *Acta Neuropathol*. 2020;140(2):231-235. doi:10.1007/s00401-020-02164-4.
2. Li H. Minimap2: pairwise alignment for nucleotide sequences. *Bioinformatics*. 2018;34(18):3094-3100. doi:10.1093/bioinformatics/bty191.
3. Robinson JT, Thorvaldsdóttir H, Winckler W, et al. Integrative genomics viewer. *Nat Biotechnol*. 2011;29(1):24-26. doi:10.1038/nbt.1754.
4. Koren S, Walenz BP, Berlin K, Miller JR, Bergman NH, Phillippy AM. Canu: scalable and accurate long-read assembly via adaptive *k*-mer weighting and repeat separation. *Genome Res*. 2017;27(5):722-736. doi:10.1101/gr.215087.116.
5. Lamark T, Johansen T. Aggrephagy: selective disposal of protein aggregates by macroautophagy. *Int J Cell Biol*. 2012;2012:736905. doi:10.1155/2012/736905.
6. Gibertini S, Ruggieri A, Cheli M, Maggi L. Protein Aggregates and Aggrephagy in Myopathies. *Int J Mol Sci*. 2023;24(9):8456. Published 2023 May 8. doi:10.3390/ijms24098456.
7. Gautier R, Douguet D, Antonny B, Drin G. HELIQUEST: a web server to screen sequences with specific alpha-helical properties. *Bioinformatics*. 2008;24(18):2101-2102. doi:10.1093/bioinformatics/btn392.
